# Astragalus-cultivated soil was a suitable bed soil for nurturing *Angelica sinensis* seedlings from the rhizosphere microbiome perspective

**DOI:** 10.1038/s41598-023-30549-4

**Published:** 2023-02-28

**Authors:** Zhi-Gang An, Feng-Xia Guo, Yuan Chen, Gang Bai, Ai-Feng Guo

**Affiliations:** 1 College of Life Science and Technology, College of Agronomy, State Key Laboratory of Aridland Crop Science, Gansu Provincial Key Lab of Good Agricultural Production for Traditional Chinese Medicine, Gansu Provincial Engineering Research Centre for Medical Plant Cultivation and Breeding, Gansu Agricultural University, Lanzhou, 730070 China; 2 Pharmacy Department, Gansu University of Chinese Medicine, Dingxi, 743000 China; 3 Jinchang Ecological Environment Bureau, Jinchang, 737100 China

**Keywords:** Plant ecology, Microbial ecology

## Abstract

*Angelica sinensis* (Oliv.) Diels is an important Chinese medicinal plant. *A*. *sinensis* seedlings are grown on an undisturbed alpine meadow soil to ensure the high-quality seedlings, but these soils are disappearing year after year. Thus, selecting a suitable bed soil for *A. sinensis* seedlings could ensure their long-term sustainability. Using HiSeq sequencing of 16S and 18S marker genes, we investigated the rhizosphere bacterial and fungal microbiotas of the seedlings grown in wheat, astragalus, potato, and angelica-cultivated soils at a geo-authentic habitat. Co-occurrence network analysis, canonical correspondence analysis, Mantel test, and Envfit test were used to examine the relationship between the microbiotas and the surrounding factors. Astragalus-cultivated soils exhibited the following properties: the highest plant weight, the highest neighborhood connectivity in the bacterial network, the highest ratio of positive/negative relationship in both bacterial and fungal networks, the highest relative abundance of the arbuscular mycorrhizal fungi and the ectomycorrhizal fungi, the lowest relative abundance of *Rhizoctonia solani*, the suitable soil pH, and the close relationship between the rhizosphere microbiotas and the ecological factors. Moreover, each growth stage has its own major drivers in all crop-cultivated soils. Climate temperature and soil pH at 56 days after planting, precipitation at 98 days, and plant weight as well as microbial biomass C and N at 129 days were the major drivers of the bacterial and fungal microbiotas. Overall, the astragalus-cultivated soil was a suitable bed soil for nurturing *A. sinensis* seedlings to replace the undisturbed alpine meadow soils.

## Introduction

*Angelica sinensis* (Oliv.) Diels (Umbelliferae) is a fragrant and herbaceous perennial plant and is widely used as a natural medicine in China. Usually, *A. sinensis* has a three-year growth cycle in a geo-authentic habitat (Dingxi, Gansu province), with the seedlings nurtured in the first year. To ensure high-quality seedlings, *A. sinensis* seedlings are traditionally cultivated in an undisturbed alpine meadow soil with rainfed agroecosystems^1^. Traditional farming methods are no longer sustainable when the number of undisturbed meadow soils decreases. Therefore, it is critical to find a cultivated soil to replace the undisturbed meadow soil.

The importance of soil selection in agricultural production has been extensively researched^2^. Researchers have identified the most suitable soils for a variety of crops in various areas. For example, American ginseng (*Panax quinquefolius* L.) was shown to be appropriate for cultivation in a maize soil of three-year continuous cropping^3^. Wang et al.^4^, Jin et al.^5^, and Bai et al.^6,7^ investigated the impacts of the cultivated farmland from a geo-authentic habitat on *A. sinensis* seedling growth.

Moreover, many studies have shown that soil properties can influence the microbial community in the soil^8^. Tkacz et al.^9^ used model plants and crop plants to show that rhizosphere microbiota was influenced by the interaction of rhizosphere type and soil composition. Rhizosphere microbiota as a subgroup of soil microorganisms is considered to be the second genome of a plant, and they are closely linked to plant growth^10^. An et al^1^ reported the characteristics of the rhizosphere bacterial and fungal communities of *A. sinensis* seedlings cultivated in an undisturbed alpine meadow soil from a geo-authentic habitat. Rhizosphere microbiota assembly is influenced by plant development^11^, soil pH^12,13^, and climate change^14^. Several previous studies have shown that different crops grown in the same soils can result in distinct responses of the soil bacterial and fungal microbiotas^3,15^. Rhizosphere microbiota are functionally diverse. Some microbes create beneficial effects on plants, such as phosphate-solubilizing and potassium-releasing^16^, protecting plants against pathogen infection^17^, N and S cycling^18,19^, and indole acetic acid production^20^, while others create harmful effects, such as plant pathogens^21^.

The rhizosphere microbiome of *A. sinensis* seedlings grown in cultivated soils from a geo-authentic habitat is currently unknown. However, building such knowledge will assist the sustainable production of this important medicinal plant. Therefore, a field experiment on the rhizosphere of *A. sinensis* seedlings was conducted in a geo-authentic habitat (Dingxi city). This study explored the bacterial and fungal microbiotas in the rhizosphere and the surrounding ecological factors, with the goal to find a suitable crop-cultivated soil for nurturing *A. sinensis* seedlings.

## Materials and methods

### Terminology and statement

The plots on which four crops had been cultivated and completed a life cycle were defined as “crop-cultivated soils”, including wheat-cultivated soils, astragalus-cultivated soils, potato-cultivated soils, and angelica-cultivated soils. All materials and methods were performed following the relevant guidelines and regulations in China.

### Study site and experimental design

The study site was located in Min County, Dingxi City, Gansu Province, China (N 34°25′27′′, E 104°28′24′′) and was 2783 m above sea level. The site is mountainous with a rainfed agroecosystem, a cool and semi-humid climate, 5–6 °C of annual average temperature, 2,219 h of yearly sunlight, 90–120 frost-free days per year, and 451.4–817.8 mm of annual precipitation.

**Figure 1:**
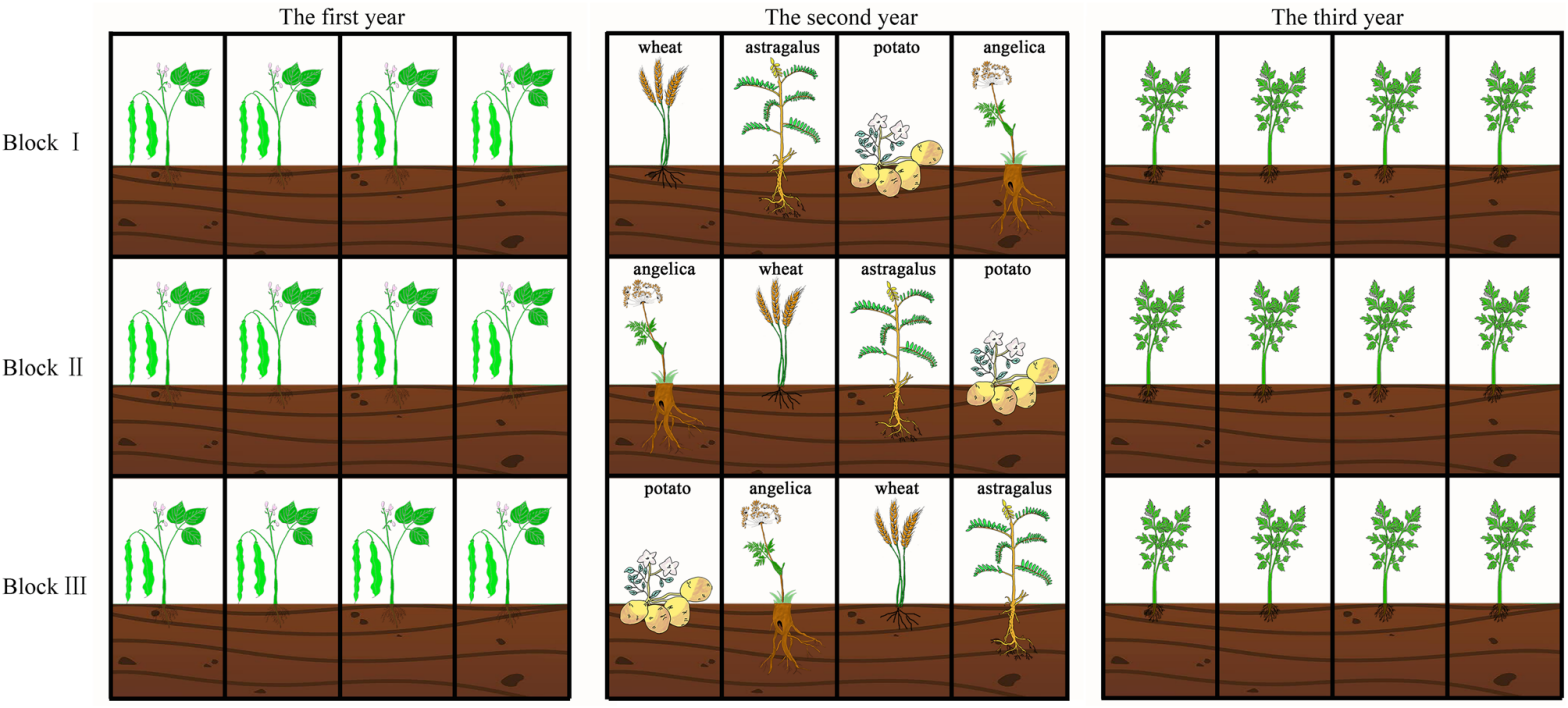
Schematic representation of the same field over time. The field was divided into three blocks. Each block contained four treatments, totaling 12 plots. The field were planted with broad bean in the first year, wheat, astragalus, potato and angelica in the second year, and *Angelica sinensis* seedlings in the third year.

In the first year, broad bean (*Vicia faba* L.) was planted in the test plot (Fig. 1). In the second year, four crops, wheat (*Triticum aestivum* L.), astragalus [*Astragalus membranaceus* (Fisch.) Bge. var. *mongholicus* (Bge.) Hsiao], potato (*Solanum tuberosum* L.), and angelica [*Angelica sinensis* (Oliv.) Diels], were respectively cultivated in a six square metre plot under a single-factor randomized block design with three replications (Fig. 1). We maintained consistent plot management after cultivating four crops, and the crops were harvested on time. In the third year, the tests of nurturing *A. sinensis* seedlings were carried out on four crop-cultivated soils (four treatments) (Fig. 1). Before sowing *A. sinensis* seeds, we applied 1.5 kg of organic fertilizers to each plot. Each plot was seeded with 10 g of seeds (thousand-seed weight of 1.63 g, seed purity of 97.7%). Each plot was covered with a 0.5 cm layer of soils. During the seedling nurturing stage, each plot was managed consistently.

### Sample preparation

In the third year, rhizosphere samples were collected from June to October during the plant growth stages of 56 days (AM), 98 days (BM), and 129 days (CM) after planting. In each plot, plant samples were comprised of five healthy plants that were randomly selected. After shaking off the loosely root-attached soils, we collected the tightly adhered rhizosphere using a sterile brush. Rhizosphere samples from the same growth stage were mixed and stored at − 80 °C. Further, the shaken off soil from the same growth stage was mixed, and the root residues were removed. After the rhizosphere samples were collected, the soil samples were used to determine the microbial biomass and the soil pH. The plants were used to determine the seedling weight.

### Ecological factors

In this study, the plant weight (PW), the soil pH (pH), the microbial biomass C (MBC), the microbial biomass N (MBN), the climate temperature (T) and the precipitation (PC) were considered ecological factors affecting the rhizosphere microbiota. The plant weight was determined by weighing a seedling without the root soils. The soil pH was measured by a glass electrode in a water-to-soil ratio of 3:1 (v/w). The microbial biomass C and N were determined by the chloroform fumigation-extraction method^22,23^ and were estimated on the oven dry soil. The test process was as follows. The soil samples were sieved (2 mm), adjusted to 40% of the water holding capacity, and incubated at 25 °C for seven days. Incubated soils that were equivalent to 10 g of the oven dry soil were transferred into a 50 mL tube and fumigated for 24 h in the dark with alcohol free CHCl_3_ (non-fumigated soils as a control group). Fumigated and non-fumigated soils were mixed with 40 mL of 0.5 M K_2_SO_4_, shaken for 30 min, and filtered with qualitative filter paper under vacuum conditions. An automated carbon and nitrogen analyzer (Multi N/C 2100s, Jena, Germany) was used to estimate the microbial biomass C and N, and the value was calculated with conversion factors of 2.20 for the microbial biomass C and 1.85 for the microbial biomass N. The information of the climate temperature and precipitation were collected from the Weather Station.

### DNA extraction, Illumina sequencing and function prediction

Total genome DNA was extracted by the cetyltrimethylammonium bromide (CTAB) method^24^. The region gene was amplified with the primer pair 515F/806R for 16S rRNA V4 and the primer pair 528F/706R for 18S rRNA V4. PCR amplification was referenced to the method described by An et al.^1^. The amplicons from each sample were mixed in equimolar amounts and then sequenced using IlluminaHiSeq (PE250, USA). Raw reads were treated by cutting off the barcodes and primer sequences, merging reads using FLASH^25^, filtering using QIIME^26^, and removing chimeras^27,28^ to obtain effective tags. Effective tags were clustered into operational taxonomic units (OTUs) using UPARSE (V 7.0.1001) with ≥ 97% sequence identity^29^. The rarefaction curves were calculated using the vegan package in R (V 3.5.0). Representative sequences in each OTU were selected, and blasted against the SILVA database (V 123) to annotate the taxonomic information for bacteria and fungi^30,31^. The multiple sequence alignment was conducted using MUSCLE (V 3.8.31)^32^. The functional prediction of microbiota was based on FAPROTAX database for bacteria^33^ and FUNGUILD database for fungi^34^. The sequences with a number of not less than one across all samples were selected and normalized to the maximum sequence count by calculating OTU relative abundances, yielding 35,042 bacterial sequences and 11,374 fungal sequences per sample.

### Co-occurrence network

Before the co-occurrence network was built, the relative abundance of OTUs performed centered log-ratio (CLR) transformation. Each feature in a sample is divided by the geometric mean of all features in this sample, and then the natural logarithm of this ratio is taken^35^. The CLR transformation can be obtained as follows:

$$CLR\left({X}_{j}\right) = \left[\mathrm{ln}\left(\frac{{X}_{1j}}{g\left({X}_{j}\right)}\right), \dots , \mathrm{ln}\left(\frac{{X}_{Dj}}{g\left({X}_{j}\right)}\right) \right]$$

where *j* is each sample, *X*_*j*_ is the list of features in a sample, *g*(*X*_*j*_) is the geometric mean of the features in sample *X*_*j*_, *X*_*1j*_ is the first feature in a sample, and *X*_*Dj*_ is the last feature in a sample of *D* values.

Co-occurrence networks were constructed based on Pearson correlation coefficients between the CLR transformed values of OTUs and the ecological factors (PW, pH, MBC, MBN, T, and PC), with a correlation coefficient − 0.60 ≥ *r* ≥ 0.60 and *P* < 0.05 (two-tailed). Network visualization was performed using CytoScape (V 3.7.2), and the neighborhood connectivity was analyzed using CytoScape's NetworkAnalyzer^36,37^.

### Statistical analyses

Ecological factors, the relative abundance of OTUs, and neighborhood connectivity were analysed using One-ANOVA with tukey’s test in Origin (2022). Mantel test (bray–curtis distance), canonical correspondence analysis, Envfit test, and functional prediction were performed using the Novogene Cloud Platform (www.novogene.com). Pathogenic microbes and mycorrhizal fungi were analysed using Kruskal–Wallis ANOVA with Dunn’s test in Origin (2022).

## Results

### Changes of ecological factors

Using One-ANOVA analyses, we investigated the effects of blocks and crop-cultivated soils on ecological factors. At AM, BM, and CM, the block had no significant effect on the plant weight, the soil pH, and the microbial biomass C and N (Supplementary Table S1). The crop-cultivated soils significantly affected the plant weight (*F* = 5.82, *P* < 0.05) and the microbial biomass C (*F* = 4.30, *P* < 0.05) at AM, the soil pH (*F* = 4.52, *P* < 0.05) at BM, and the plant weight (*F* = 11.35, *P* < 0.01) and the microbial biomass C (*F* = 4.15, *P* < 0.05) at CM (Table 1). In all crop-cultivated soils at CM, astragalus-cultivated soils had the highest plant weight, followed by wheat, angelica and potato-cultivated soils (Table 1).

**Table 1:** Effect of crop-cultivated soils on ecological factors during the growth stage.

Factors	Stages	Crop-cultivated soils			
		Wheat	Astragalus	Potato	Angelica
PW/(g)	AM	0.16 ± 0.02a	0.07 ± 0.04b	0.06 ± 0.03b	0.10 ± 0.04b
	BM	0.63 ± 0.16a	0.46 ± 0.02a	0.51 ± 0.24a	0.59 ± 0.12a
	CM	1.51 ± 0.15a	1.76 ± 0.38a	0.82 ± 0.16b	1.02 ± 0.08b
pH	AM	8.33 ± 0.16a	8.40 ± 0.02a	8.38 ± 0.05a	8.42 ± 0.07a
	BM	8.00 ± 0.06ab	7.94 ± 0.03b	8.14 ± 0.14a	8.10 ± 0.03a
	CM	7.91 ± 0.32a	8.06 ± 0.05a	8.11 ± 0.19a	8.49 ± 0.45a
MBC/(mg kg^-1^)	AM	109.67 ± 37.92b	178.24 ± 11.19a	156.38 ± 12.85ab	181.30 ± 36.42a
	BM	404.06 ± 80.88a	410.81 ± 37.85a	410.23 ± 47.18a	430.90 ± 69.17a
	CM	334.71 ± 13.97b	346.19 ± 29.91b	381.89 ± 55.66ab	434.15 ± 39.84a
MBN/(mg kg^-1^)	AM	48.37 ± 7.93a	54.52 ± 7.77a	51.48 ± 7.90a	52.90 ± 5.00a
	BM	87.39 ± 15.71a	90.29 ± 0.24a	82.36 ± 2.93a	88.81 ± 14.91a
	CM	75.72 ± 12.55a	80.50 ± 6.33a	83.40 ± 10.10a	92.17 ± 4.08a
T/(℃)	AM	17.33 ± 1.78			
	BM	14.43 ± 3.47			
	CM	9.55 ± 1.90			
PC/(mm d^-1^)	AM	2.64 ± 0.07			
	BM	4.47 ± 0.47			
	CM	2.11 ± 0.08			

Data are presented as standard deviation (SD), n = 3. Different lowercase letters represented statistically significant differences among crop-cultivated soils by One-ANOVA with Tukey’s test at *P* < 0.05. AM, BM, and CM represented the different growth stages at 56 days, 98 days, and 129 days respectively.

*PW* the plant weight, *pH* the soil pH, *MBC* the microbial biomass C, *MBN* the microbial biomass N, *T* the climate temperature, *PC* the precipitation.

### Co-occurrence networks

A total of 1,492,560 bacterial reads and 1,145,232 fungal reads were obtained from 36 samples. After filtering, 1,261,512 bacterial and 409,464 fungal reads were used in the study. In the rarefaction curves, the number of sequencing data provided sufficient information on microbial diversity (Supplementary Fig. S1). A total of 616 OTUs, 585 bacterial and 31 fungal, were obtained from 36 samples.

**Figure 2:**
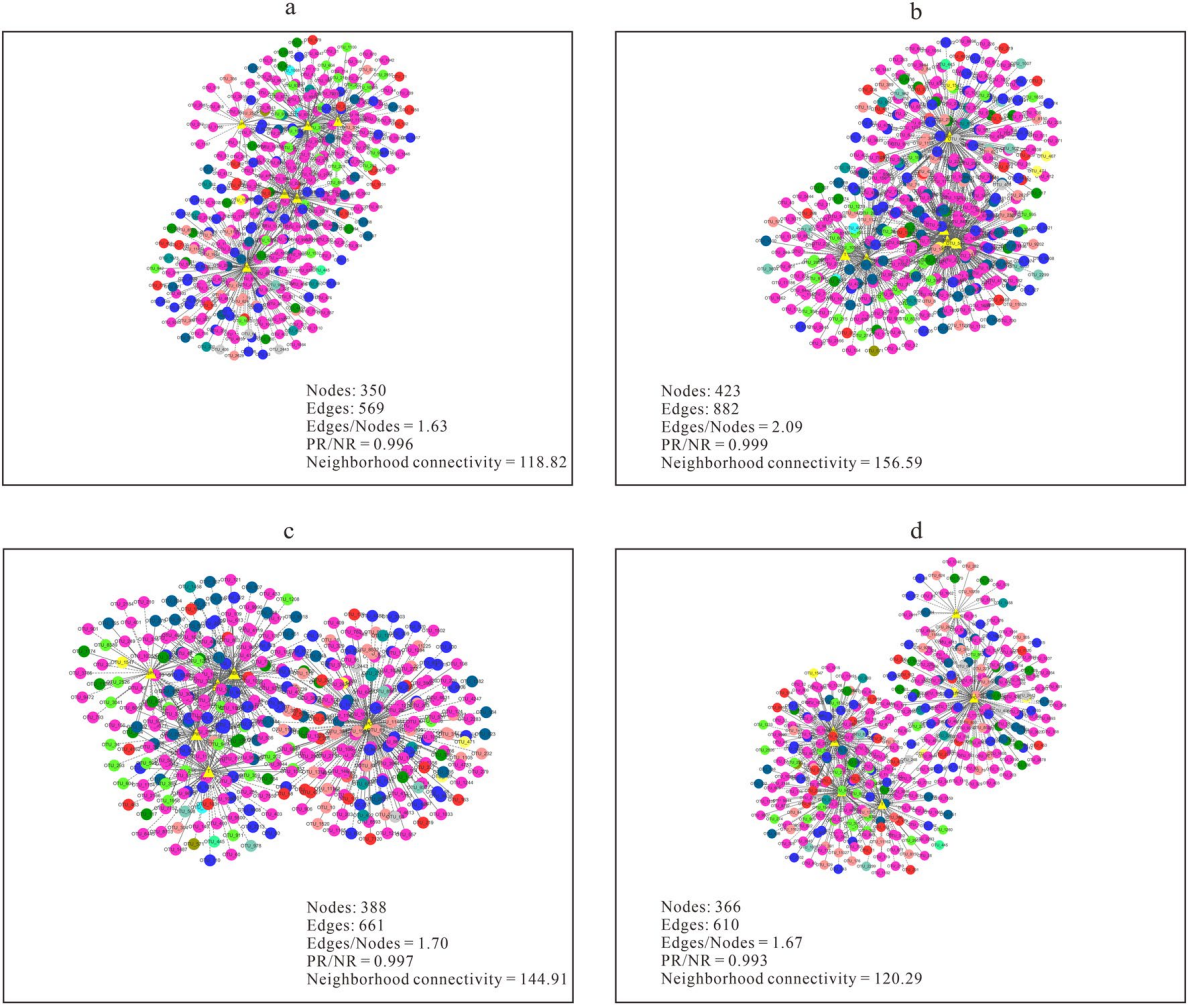
Co-occurrence networks between bacterial OTUs and ecological factors (PW, pH, MBC, MBN, T, and PC) in wheat-cultivated soils (**a**), astragalus-cultivated soils (**b**), potato-cultivated soils (**c**) and angelica-cultivated soils (**d**). The filled colors in nodes indicate phylum level, and the filled yellow color in triangles indicates ecological factors. The solid lines are a positive relationship, and the dashed lines are a negative relationship. Nodes, the number of nodes in a network; edges, the number of edges in a network; edges/nodes, the ratio of edges to nodes; PR, the number of positive relationships; NR, the number of negative relationships; PR/NR, the ratio of PR to NR; neighborhood connectivity, the average neighborhood connectivity of a network. The order of neighborhood connectivity was astragalus^(a)^ > potato^(b)^ > angelica^(c)^ > wheat^(c)^, with superscript lowercase letters in parentheses representing statistically significant differences under One-ANOVA with Tukey’s test at *P* < 0.05.

In order to better understand the interactions between OTUs and ecological parameters, co-occurrence networks between them were examined throughout the growth time-series. Neighborhood connectivity of a network is used to illustrate its complexity. The effect of crop-cultivated soils on neighborhood connectivity was analyzed by One-ANOVA. The results showed that crop-cultivated soils significantly affected the neighborhood connectivity of bacterial networks (*F* = 57.690, *P* < 0.05) (Fig. 2), but did not significantly affect the neighborhood connectivity of fungal networks (*F* = 1.998, *P* > 0.05) (Supplementary Fig. S2). In the bacterial networks (Fig. 2), the neighborhood connectivity of astragalus-cultivated soils was higher than that of other soils. In the bacterial networks of astragalus-cultivated soils, the edges/nodes ratio of 2.09 was higher than that of other soils, and the positive/negative relationship ratio of 0.999 was also higher than that of other soils. These results showed that the bacterial microbiota in astragalus-cultivated soils was closely related to the ecological factors. Moreover, in the fungal networks of astragalus-cultivated soils (Supplementary Fig. S2), the positive/negative relationship ratio of 1.26 was higher than that of other soils. In terms of neighborhood connectivity, bacteria were more sensitive than fungi to the changes in the crop-cultivated soils.

### Pathogenic microbes and mycorrhizal fungi

FAPROTAX and FUNGUILD databases were used to predict pathogenic microbes and mycorrhizal fungi in the microbiotas. Using Kruskal–Wallis ANOVA with Dunn’s test, crop-cultivated soils obviously affect the relative abundance of arbuscular mycorrhizal fungi (*H* = 8.228, *P* < 0.05) and ectomycorrhizal fungi (*H* = 12.484, *P* < 0.05) (Supplementary Table S2). The relative abundance of arbuscular mycorrhizal fungi in astragalus-cultivated soils was significantly higher than that in angelica-cultivated soils, and the same result was observed for ectomycorrhizal fungi in astragalus-cultivated soils. Astragalus-cultivated soils had the highest relative abundance of arbuscular mycorrhizal fungi and ectomycorrhizal fungi in all crop-cultivated soils. Additionally, we found that *Rhizoctonia solani*, one of the pathogens associated with *A. sinensis*, occupied all crop-cultivated soils throughout the growth stage, with the lowest average relative abundance in astragalus-cultivated soils.

### Drivers of microbial communities

Mantel test provides a means to test the correlation of multivariate data and is widely used in ecological studies. The correlations between the microbiotas and the ecological factors were investigated by the Mantel test (Tables 2 and 3). The bacterial and fungal microbiotas were significantly correlated with the plant weight in all crop-cultivated soils, showing that the bacterial and fungal microbiotas were intimately related to the seedling growth. In the study, only the pH of astragalus-cultivated soils was significantly related to both the bacterial and fungal microbiotas, implying that this soil pH may facilitate the assembly of the bacterial and fungal microbiotas. Except for the precipitation, all other ecological factors in astragalus-cultivated soils were significantly correlated with the bacterial and fungal microbiotas.

**Table 2:** Correlations between bacterial microbiota and ecological factors in crop-cultivated soils.

Crop-cultivated soils	PW/(g)		pH		MBC/(mg kg^-1^)		MBN/(mg kg^-1^)		T/(℃)		PC/(mm d^-1^)	
	*r*	*P*	*r*	*P*	*r*	*P*	*r*	*P*	*r*	*P*	*r*	*P*
Wheat	0.66	0.004**	0.28	0.092	0.51	0.011*	0.42	0.01*	0.48	0.011	0.28	0.075
Astragalus	0.67	0.005**	0.66	0.004**	0.57	0.01*	0.60	0.008**	0.37	0.03*	0.19	0.134
Potato	0.44	0.014*	0.16	0.179	0.26	0.071	0.28	0.062	0.36	0.025*	0.35	0.03*
Angelica	0.66	0.001**	0.18	0.179	0.39	0.027*	0.48	0.011*	0.41	0.017*	0.25	0.093

Asterisk represented statistically significant differences between bacterial microbiota and ecological factors by Mantel test at *P* < 0.05 (*) and 0.01 (**). The meanings of PW, pH, MBC, MBN, T and PC were illustrated in Table 1. *r*, the correlation of two matrices.

**Table 3:** Correlations between fungal microbiota and ecological factors based in crop-cultivated soils.

Crop-cultivated soils	PW/(g)		pH		MBC/(mg kg^-1^)		MBN/(mg kg^-1^)		T/(℃)		PC/(mm d^-1^)	
	*r*	*P*	*r*	*P*	*r*	*P*	*r*	*P*	*r*	*P*	*r*	*P*
Wheat	0.63	0.004**	0.22	0.149	0.77	0.002**	0.40	0.027*	0.32	0.05	0.09	0.274
Astragalus	0.66	0.004**	0.56	0.01*	0.54	0.011*	0.48	0.013*	0.37	0.026*	0.10	0.228
Potato	0.61	0.003**	0.37	0.056	0.59	0.015**	0.53	0.013**	0.34	0.048*	0.20	0.099
Angelica	0.71	0.001**	0.10	0.237	0.38	0.023*	0.46	0.011*	0.33	0.041*	0.05	0.359

Asterisk represented statistically significant differences between fungal microbiota and ecological factors by Mantel test at *P* < 0.05 (*) and 0.01 (**). The meanings of PW, pH, MBC, MBN, T and PC were illustrated in Table 1. *r*, the correlation of two matrices.

**Figure 3:**
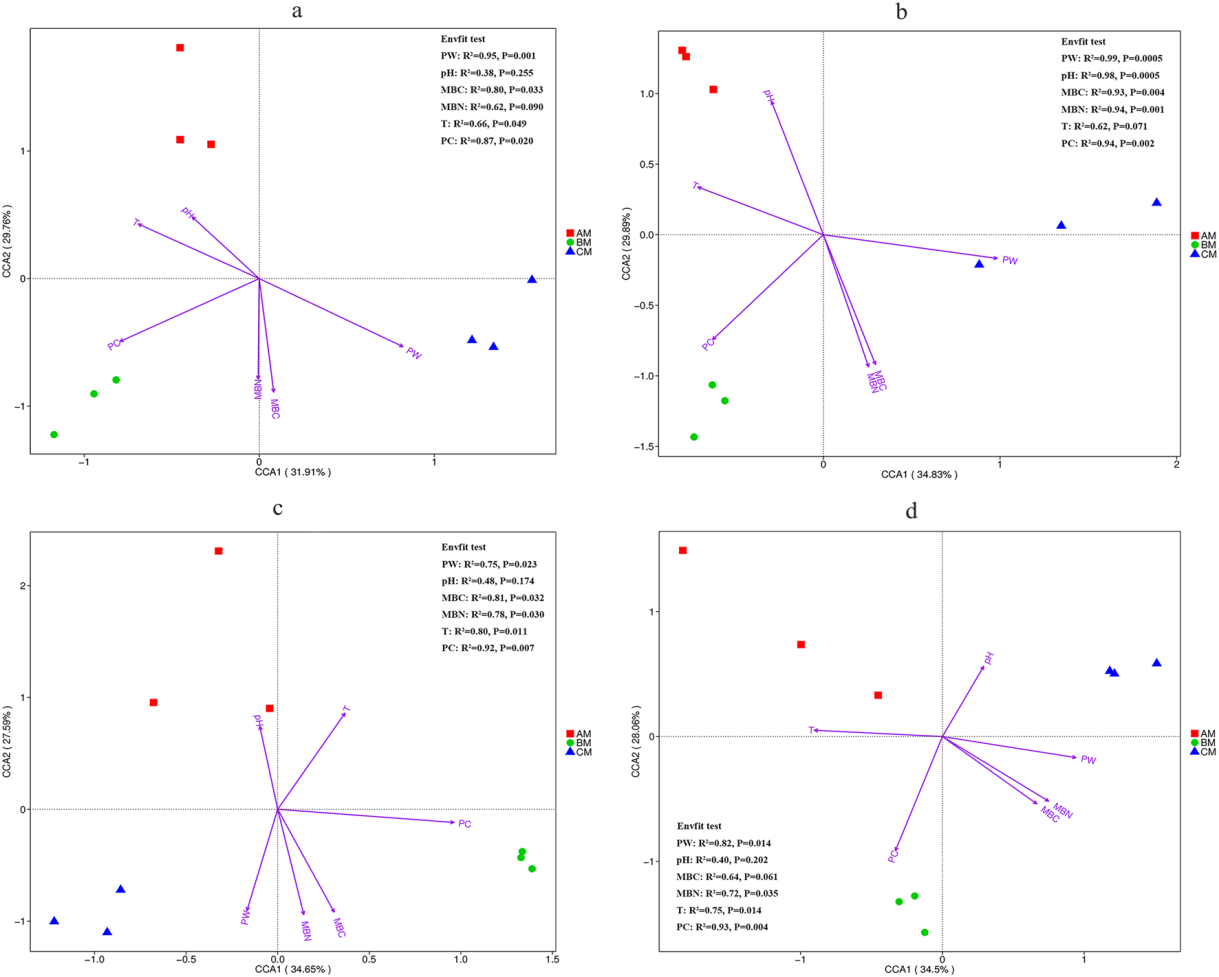
Canonical correspondence analysis between bacterial microbiota and ecological factors (PW, pH, MBC, MBN, T, and PC) in wheat-cultivated soils (**a**), astragalus-cultivated soils (**b**), potato-cultivated soils (**c**), and angelica-cultivated soils (**d**). AM, BM, and CM represented the different growth stages at 56 days, 98 days (AM), and 129 days respectively. In Envfit test results: R^2^, the coefficient of determination between factors and species distribution; P, the statistical difference.

Canonical correspondence analysis is a nonlinear multivariate direct gradient analysis method in ecological studies. This method can easily determine the causal relationships between species distributions and environmental variables. In this study, it was used to assess the association between bacterial and fungal microbiotas and ecological factors. We found that each growth stage has its own major drivers in all crop-cultivated soils (Fig. 3 and Supplementary Fig. S3). Climate temperature and soil pH at AM, precipitation at BM, and plant weight as well as microbial biomass C and N at CM were the major drivers of the bacterial microbiotas (Fig. 3). Similar findings were also found in the fungal microbiotas (Supplementary Fig. S3).

Envfit test was used to identify the environmental factors that significantly affected the microbial communities. The results described the drivers for the assembly of bacterial and fungal microbiotas (Fig. 3 and Supplementary Fig. S3). For all crop-cultivated soils, the plant weight and precipitation were two significant factors affecting the assembly for the bacterial microbiota (Fig. 3), while the microbial biomass C was only one significant factor for the fungal microbiota (Supplementary Fig. S3). These results indicated that the bacterial microbiota was more sensitive to ecological factors than the fungal microbiota. Additionally, the factors that significantly affected the assembly of the bacterial and fungal microbiotas from a crop-cultivated soil were distinct. We found that more ecological factors that influenced the bacterial and fungal microflora in astragalus-cultivated soils, demonstrating a closer relationship between the microbiotas and the ecological factors in astragalus-cultivated soils.

## Discussion

Properties of crop-cultivated soils are affected by the planting management of previous crops. It is well known that when former plants complete a life cycle, they can lead to soil properties that differ from bulk soils^38–40^. Different soil properties resulted in variations in soil basic fertility, which in turn led to differences in soil ecological factors. In this study, crop-cultivated soils at different growth stages differently impacted the ecological factors of the rhizosphere microbiota. The seedling weight in astragalus-cultivated soils was higher than that of other soils. Because the root system and growth power of seedlings were strong and the root disease incidence and disease index were low, which was discussed by Jin et al.^5^. Bai et al.^6^ explained the advantages of pea-astragalus alternate soil for cultivating high-quality angelica seedlings from the relative conductivity, amino acid leakage rate, automatic oxidation rate, soluble protein content, sugar content, malondialdehyde content as well as superoxide dismutase, peroxidase and catalase activities.

In the study, the number of bacterial OTUs was almost six times higher than that of fungal OTUs, and similar results in other plants had been reported^41,42^. Network analysis based on the correlation between factors is widely used in soil microbial ecology^43^, for example, between soil physicochemical properties and bacterial communities^44^, and between plant growth age and microbial communities^45^. Network analysis could help researchers understand that the microbiome in complex systems was influenced by the gradient changes in ecological factors, and infer the assembly rules of microbial communities. However, there is a lack of theoretical models to explain complex relationships between organisms using network diagram parameters^46^. In this study, these correlations between OTUs and factors may reflect the changes in microbial ecological behavior (niche) caused by specific soil-driven environmental variations^47,48^. Chamberlain et al.^13^ found that corn-soybean rotation resulted in variations in nutrient availability, soil organic matter content and pH, and that had an impact on the structure of bacterial communities in the bulk soil, which differed from continuously cropped corn and soybean. Based on our findings, we concluded that crop-cultivated soils distinctly affected the interactions between the microbiota and the ecological factors. Using the neighborhood connectivity as an indicator of network complexity, the bacterial network of astragalus-cultivated soils was more complex than that of other soils. The stability of this network may be higher than that of other networks^49^. Therefore, this bacterial microbiota may have a greater ability to resist external disturbances^50,51^.

Previous studies showed that root border cells, root exudates, and root deposits act as nutrient substances that recruit the members of rhizosphere microbiota^52–54^, which could explain our finding that the rhizosphere microbiota was closely associated with the plant weight. The similar result was also found in a new study, reporting that the rhizosphere microbial community composition of *Medicago sativa* was significantly correlated with *M*. *sativa* biomass^55^. By analyzing the results of Mantel test, we concluded that among crop-cultivated soils, the bacterial and fungal microbiotas in astragalus-cultivated soils were most closely related to their ecological factors.

In the study, the major drivers of the bacterial and fungal microbiotas in each crop-cultivated soil were different, which may be related to soil properties caused by the previous crop growth process^56^. This mean that there is more environmentally-driven ecological variance in these plots. Previous studies have also reported that crop rotation or former crop cultivation had influences on soil microbial biomass^57^, enzyme activities^58^, and microbial microbiotas^59^. The pH is one of the most important soil properties. Many studies have shown that soil pH is a major driver for the assembly of soil bacterial and fungal microbiotas^60–62^. Soil pH was the major driver in the early growth stage, which should be an important factor to be considered when choosing the bed soil for the seedling growth. For example, soil pH was assessed in the farmland of tomato^63^, maize^64^ and haskap^60^. In our study, only the pH of astragalus-cultivated soils was shown to be closely connected to the bacterial and fungal microbiotas, as confirmed by Mantel test and Envfit test. Therefore, the pH of astragalus-cultivated soils was possibly beneficial to the assembly of the bacterial and fungal microbiotas. The location where the seedlings were nurtured is a rainfed agroecosystem. The results showed that precipitation was the main driver of bacterial and fungal microbiotas in the middle growth stage. Many studies have found that the amount of water irrigation affects the structure of soil microbiotas^64,65^. Previous studies showed that soil microbes were more responsive to soil management^66^, and their biomass as reservoirs of soil C and N parameters was an important indicator in soils^67^. Soil microbial biomass can be regulated by fertilization practices^68,69^. Tan et al.^70^ found that the soil microbial biomass C in a peanut cropping system was significantly increased by applying biochar plus organic fertilizer. Liu, et al.^71^ and Li et al.^72^ found that microbial biomass N was enhanced by N fertilization.

Additionally, in terms of the network neighborhood connectivity and Envfit test, we found that the bacterial microbiota was more sensitive to the changes of the crop-cultivated soils than the fungal microbiota. This differential response could be due to the distinction in soil moisture^73^, soil organic carbon^74^ or root activity^42^. The balance between beneficial and harmful microbes in rhizosphere is one of the important factors affecting plant health^75^. Many studies reported the mycorrhizal fungi on legume plants^76,77^. Astragalus-cultivated soils were characterized by the lower relative abundance of the pathogenic bacteria and fungi as well as the higher relative abundance of the arbuscular mycorrhizal fungi and ectomycorrhizal fungi. This microbial ecology environment may be more beneficial to the seedling growth^78,79^.

## Conclusion

We investigated the rhizosphere microbiome of *A. sinensis* seedlings cultivated in four crop-cultivated soils at a geo-authentic habitat. Overall, astragalus-cultivated soils exhibited the following properties: the highest plant weight, the highest neighborhood connectivity in the bacterial network, the highest ratio of positive/negative relationship in both bacterial and fungal networks, the highest relative abundance of the ectomycorrhizal fungi and the arbuscular mycorrhizal fungi, the lowest relative abundance of *R. solani*, the suitable soil pH, and the close relationship between the rhizosphere microbiotas and the ecological factors. Therefore, the astragalus-cultivated soil was a suitable bed soil for nurturing *A. sinensis* seedlings to replace the undisturbed alpine meadow soils. The study increased the understanding of the rhizosphere microbiome of *A. sinensis* seedlings at a geo-authentic habitat.

## Supplementary Information

Supplementary Information.

## Data Availability

The raw data of the sequence had been deposited into the NCBI Short Read Archive under accession PRJNA720350 (https://www.ncbi.nlm.nih.gov/bioproject/PRJNA720350).
