# Supplementary material for: Astragalus-cultivated soil was a suitable bed soil for nurturing Angelica sinensis seedlings from the rhizosphere microbiome perspective
Source: Sci Rep. 2023 Feb 28;13:3388. doi: 10.1038/s41598-023-30549-4 (PMC9974959; doi:10.1038/s41598-023-30549-4)
Supplement: Supplementary file 1 — Supplementary Information. [file 41598_2023_30549_MOESM1_ESM.zip › Supplementary material/Supplementary Figure S1 caption.pdf]

Fig. S1 Rarefaction curves of bacterial microbiota (a) and fungal microbiota (b). AM, BM, and CM represented the different growth stages at 56 days, 98 days, and 129 days respectively. 11, 12 and 13, three replications of wheat-cultivated soils; 21, 22 and 23, three replications of astragalus-cultivated soils; 31, 32 and 33, three replications of potato-cultivated soils; 41, 42 and 43, three replications of angelica-cultivated soils.
