# Supplementary material for: Astragalus-cultivated soil was a suitable bed soil for nurturing Angelica sinensis seedlings from the rhizosphere microbiome perspective
Source: Sci Rep. 2023 Feb 28;13:3388. doi: 10.1038/s41598-023-30549-4 (PMC9974959; doi:10.1038/s41598-023-30549-4)
Supplement: Supplementary file 1 — Supplementary Information. [file 41598_2023_30549_MOESM1_ESM.zip › Supplementary material/Supplementary Figure S2 caption.pdf]

Fig. S2 Co-occurrence networks between fungal OTUs and ecological factors (PW, pH, MBC, MBN, T, and PC) in wheat-cultivated soils (a), astragalus-cultivated soils (b), potato-cultivated soils (c) and angelica-cultivated soils (d). The filled colors in nodes indicate phylum level, and the filled yellow color in triangles indicates ecological factors. The solid lines are a positive relationship, and the dashed lines are a negative relationship. Nodes, the number of nodes in a network; edges, the number of edges in a network; edges/nodes, the ratio of edges to nodes; PR, the number of positive relationships; NR, the number of negative relationships; PR/NR, the ratio of PR to NR; neighborhood connectivity, the average neighborhood connectivity of a network. The order of neighborhood connectivity was angelica$^{(a)}$ > potato$^{(a)}$ > wheat$^{(a)}$ > astragalus$^{(a)}$, with superscript lowercase letters representing statistically significant differences under One-ANOVA with Tukey's test at $P < 0.05$.
