# Supplementary material for: Astragalus-cultivated soil was a suitable bed soil for nurturing Angelica sinensis seedlings from the rhizosphere microbiome perspective
Source: Sci Rep. 2023 Feb 28;13:3388. doi: 10.1038/s41598-023-30549-4 (PMC9974959; doi:10.1038/s41598-023-30549-4)
Supplement: Supplementary file 1 — Supplementary Information. [file 41598_2023_30549_MOESM1_ESM.zip › Supplementary material/Supplementary Figure S3 caption.pdf]

Fig. S3 Canonical correspondence analysis between fungal microbiota and ecological factors (PW, pH, MBC, MBN, T, and PC) in wheat-cultivated soils (a), astragalus-cultivated soils (b), potato-cultivated soils (c), and angelica-cultivated soils (d). AM, BM, and CM represented the different growth stages at 56 days, 98 days, and 129 days respectively. In Envfit test results: R^2, the coefficient of determination between factors and species distribution; P, the statistical difference.
