# Supplementary material for: Astragalus-cultivated soil was a suitable bed soil for nurturing Angelica sinensis seedlings from the rhizosphere microbiome perspective
Source: Sci Rep. 2023 Feb 28;13:3388. doi: 10.1038/s41598-023-30549-4 (PMC9974959; doi:10.1038/s41598-023-30549-4)
Supplement: Supplementary file 1 — Supplementary Information. [file 41598_2023_30549_MOESM1_ESM.zip › Supplementary material/Supplementary Table S1.pdf]

Table S1 Effects of the block groups on ecological factors during the growth stage

Stages	Factors	Blocks		
		Block Ⅰ	Block Ⅱ	Block Ⅲ
AM	PW/(g)	0.11 ± 0.04a	0.07 ± 0.05a	0.12 ± 0.06a
	pH	8.37 ± 0.13a	8.40 ± 0.05a	8.37 ± 0.09a
	MBC/($mg \cdot kg^{-1}$)	147.87 ± 32.04a	150.27 ± 54.92a	171.05 ± 29.00a
	MBN/($mg \cdot kg^{-1}$)	56.47 ± 6.47a	49.99 ± 7.40a	49.00 ± 4.51a
BM	PW/(g)	0.48 ± 0.15a	0.60 ± 0.16a	0.58 ± 0.16a
	pH	8.03 ± 0.07a	8.05 ± 0.03a	8.05 ± 0.17a
	MBC/($mg \cdot kg^{-1}$)	377.63 ± 59.94a	467.84 ± 48.08a	396.50 ± 69.57a
	MBN/($mg \cdot kg^{-1}$)	83.74 ± 9.44a	93.78 ± 10.13a	84.12 ± 8.72a
CM	PW/(g)	1.43 ± 0.59a	1.24 ± 0.50a	1.17 ± 0.24a
	pH	8.00 ± 0.32a	8.25 ± 0.48a	8.19 ± 0.17a
	MBC/($mg \cdot kg^{-1}$)	387.71 ± 54.09a	354.67 ± 26.35a	380.34 ± 73.65a
	MBN/($mg \cdot kg^{-1}$)	79.51 ± 14.22a	81.61 ± 7.40a	87.74 ± 6.96a

Note: data are presented as standard deviation (SD), n = 4. Different lowercase letters represented statistically significant differences among block groups by One-ANOVA with Tukey's test at $P < 0.05$.
