# Supplementary material for: Astragalus-cultivated soil was a suitable bed soil for nurturing Angelica sinensis seedlings from the rhizosphere microbiome perspective
Source: Sci Rep. 2023 Feb 28;13:3388. doi: 10.1038/s41598-023-30549-4 (PMC9974959; doi:10.1038/s41598-023-30549-4)
Supplement: Supplementary file 1 — Supplementary Information. [file 41598_2023_30549_MOESM1_ESM.zip › Supplementary material/Supplementary Table S2.pdf]

Table S2 Effect of crop-cultivated soils on the relative abundance of pathogenic microbes and mycorrhizal fungi across all growth stage

Microbes	Crop-cultivated soils			
	Wheat	Astragalus	Potato	Angelica
Bacterial pathogen	0.0017 ± 0.0014a	0.0015 ± 0.0010a	0.0012 ± 0.0005a	0.0017 ± 0.0015a
Fungal pathogen	0.1425 ± 0.0670a	0.1258 ± 0.0386a	0.1015 ± 0.0364a	0.1275 ± 0.0655a
Arbuscular mycorrhizal fungi	0.0157 ± 0.0068ab	0.0281 ± 0.0176a	0.0153 ± 0.0083ab	0.0102 ± 0.0032b
Ectomycorrhizal fungi	0.0057 ± 0.0013abc	0.0086 ± 0.0052a	0.0080 ± 0.0045ab	0.0024 ± 0.0023c
Rhizoctonia solani	0.0050 ± 0.0134a	0.0006 ± 0.0007a	0.0012 ± 0.0023a	0.0023 ± 0.0056a

Note: data are presented as standard deviation (SD), n = 9. Different lowercase letters represented statistically significant differences among crop-cultivated soils by Kruskal-Wallis ANOVA with Dunn's test at $P < 0.05$.
