# Supplementary figures and images for: Astragalus-cultivated soil was a suitable bed soil for nurturing Angelica sinensis seedlings from the rhizosphere microbiome perspective
Source: Sci Rep. 2023 Feb 28;13:3388. doi: 10.1038/s41598-023-30549-4 (PMC9974959; doi:10.1038/s41598-023-30549-4)

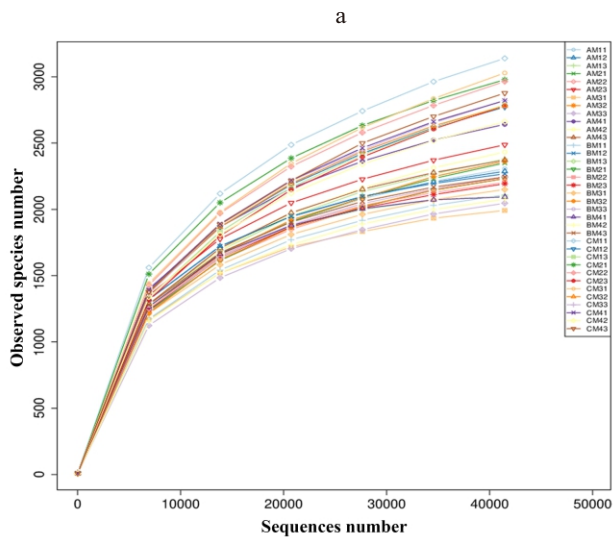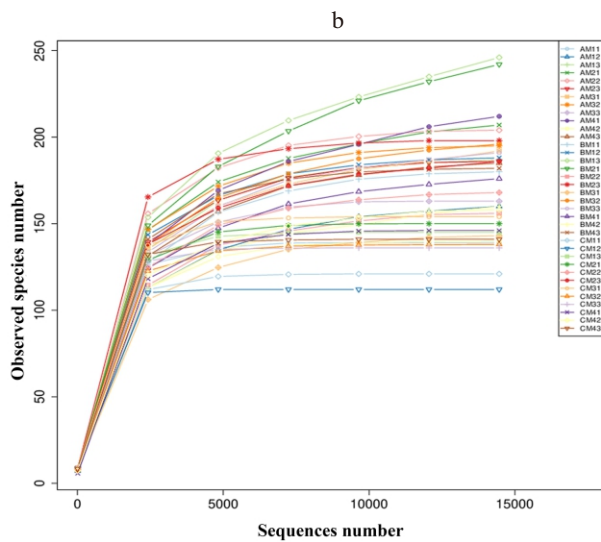

Supplement: Supplementary file 1 — Supplementary Information. [file 41598_2023_30549_MOESM1_ESM.zip › Supplementary material/Supplementary Figure S1.pdf]

a

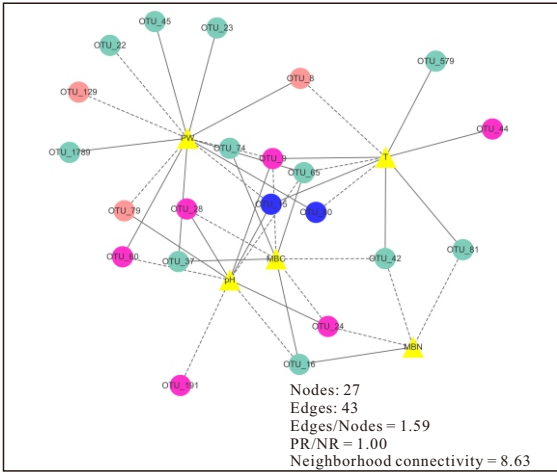

b

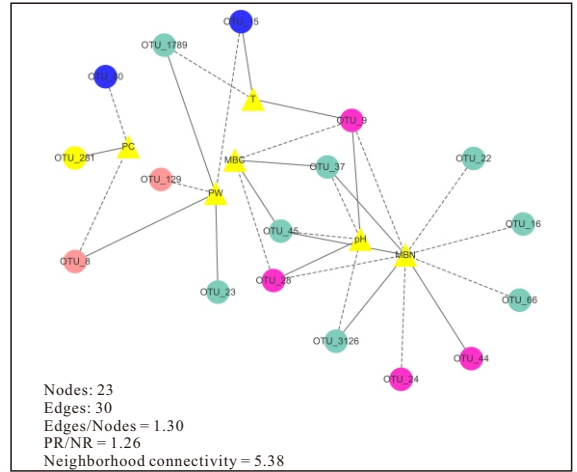

c

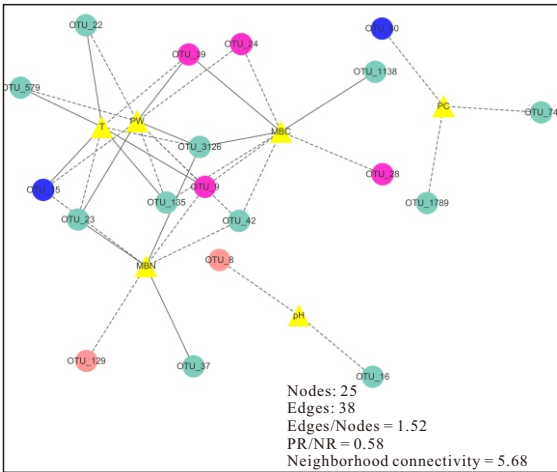

d

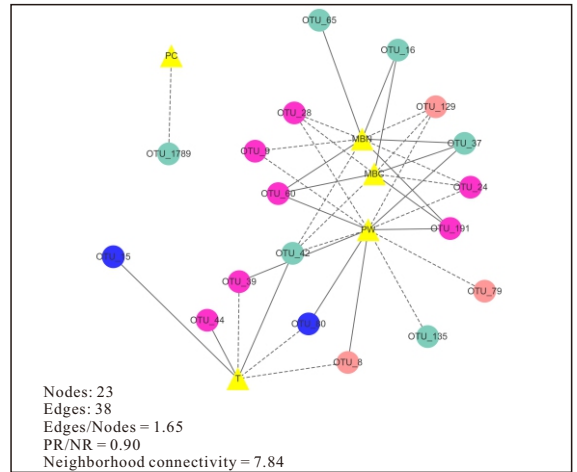

Supplement: Supplementary file 1 — Supplementary Information. [file 41598_2023_30549_MOESM1_ESM.zip › Supplementary material/Supplementary Figure S2.pdf]

a

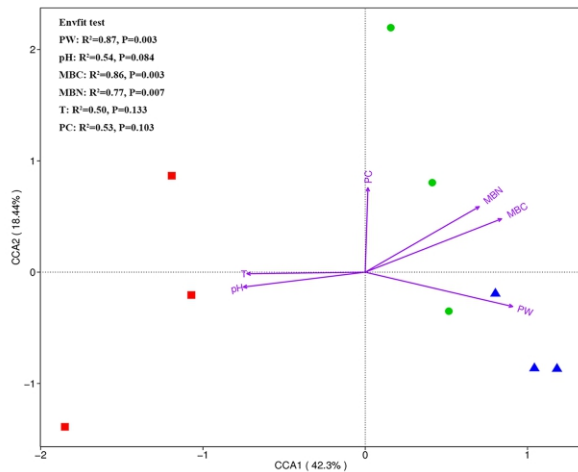

b

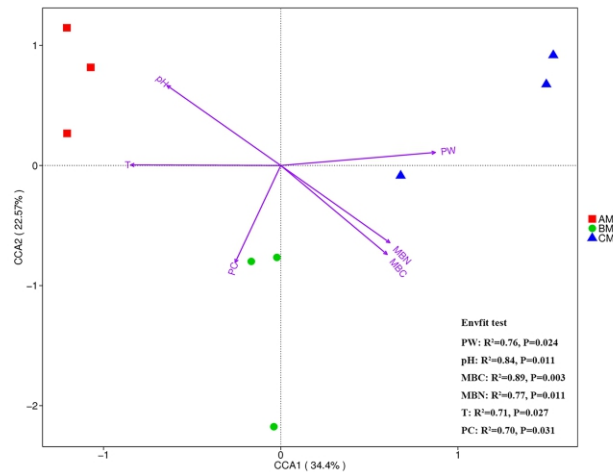

c

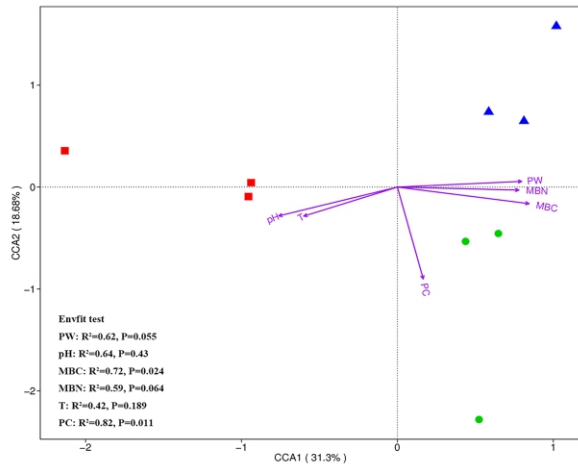

d

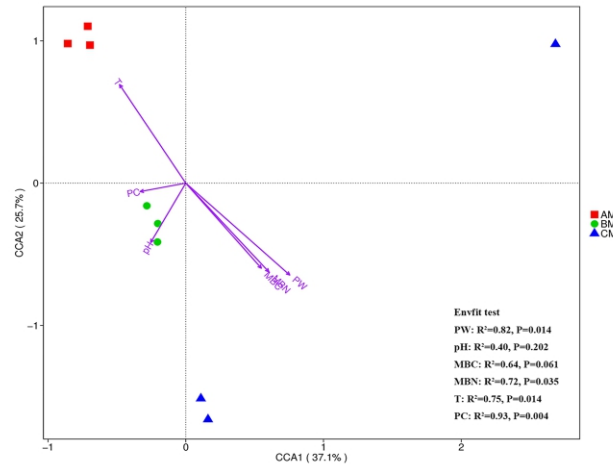

Supplement: Supplementary file 1 — Supplementary Information. [file 41598_2023_30549_MOESM1_ESM.zip › Supplementary material/Supplementary Figure S3.pdf]
